# Low Basicity as a Characteristic for Atypical Ligands of Serotonin Receptor 5-HT2

**DOI:** 10.3390/ijms22031035

**Published:** 2021-01-21

**Authors:** Sabina Podlewska, Ryszard Bugno, Enza Lacivita, Marcello Leopoldo, Andrzej J. Bojarski, Jadwiga Handzlik

**Affiliations:** 1 Department of Technology and Biotechnology of Drugs, Faculty of Pharmacy, Jagiellonian University Medical College, 9 Medyczna Street, 30-688 Kraków, Poland; 2 Maj Institute of Pharmacology, Polish Academy of Sciences, 12 Smętna Street, 31-343 Kraków, Poland; bugno@if-pan.krakow.pl (R.B.); bojarski@if-pan.krakow.pl (A.J.B.); 3 Department of Pharmacy-Drug Sciences, University of Bari “Aldo Moro”, via E. Orabona 4, 70125 Bari, Italy; enza.lacivita@uniba.it (E.L.); marcello.leopoldo@uniba.it (M.L.)

**Keywords:** serotonin receptors, docking, G protein-coupled receptors, nonbasic ligands, structure-based drug design

## Abstract

Serotonin receptors are extensively examined by academic and industrial researchers, due to their vital roles, which they play in the organism and constituting therefore important drug targets. Up to very recently, it was assumed that the basic nitrogen in compound structure is a necessary component to make it active within this receptor system. Such nitrogen interacts in its protonated form with the aspartic acid from the third transmembrane helix (D3x32) forming a hydrogen bond tightly fitting the ligand in the protein binding site. However, there are several recent studies that report strong serotonin receptor affinity also for compounds without a basic moiety in their structures. In the study, we carried out a comprehensive in silico analysis of the low-basicity phenomenon of the selected serotonin receptor ligands. We focused on the crystallized representatives of the proteins of 5-HT_1B_, 5-HT_2A_, 5-HT_2B_, and 5-HT_2C_ receptors, and examined the problem both from the ligand- and structure-based perspectives. The study was performed for the native proteins, and for D3x32A mutants. The investigation resulted in the determination of nonstandard structural requirements for activity towards serotonin receptors, which can be used in the design of new nonbasic ligands.

## 1. Introduction

Serotonin receptors (5-HTRs) are a group of proteins extensively examined by academic and commercial researchers, due to their vital roles, which they play in the organism, constituting thus important drug targets for a wide range of disorders [1,2]. Their endogenous ligand, serotonin, modulates numerous processes, such as mood, perception, reward, anger, aggression, appetite, memory, sexuality, attention, among others; however, it also controls other, non-neuropsychological processes, such as vasoconstriction, digestion, muscle contraction, and inflammatory mediation [1,3,4,5,6,7]. The 5-HT receptors can be found both in the central and peripheral nervous system—their vast majority (≈90%) is located in the gastrointestinal tract, several percent of serotonin belongs to platelets, and around 1–2% of serotonin receptors present in the human body is located in the central nervous system (CNS) [8]. Still, serotonin receptors are mainly considered as targets for CNS-related disorders, such as depression, anxiety, Alzheimer’s disease, schizophrenia, cognitive disorders, bipolar disorder, etc. [9,10]. Examples of non-CNS applications of drugs targeting the 5-HT system include gastroprokinetic drugs (such as Tegaserod, a 5-HT_4_R agonist [11]), but there is also evidence for the role of 5-HTR agonists in the arterial blood pressure control [12,13] and regulation of inflammatory response [14]. 

The current classification of serotonin receptors group them into seven classes (5-HT_1-7_) with a total of 14 members. Except for 5-HT_3_R which is a ligand-gated ion channel, serotonin receptor actions are mediated via G proteins, which makes them representatives of the G protein-coupled receptors (GPCRs) [2,15,16]. All metabotropic 5-HTRs share the same architecture of seven transmembrane domains (TMDs) and one intramembrane helix 8 (H8), which are connected by intra- and extracellular loops (ICLs and ECLs, respectively). As their natural ligand, serotonin is a biogenic amine neurotransmitter, serotonin receptors belong to the class of aminergic GPCRs.

The desire to find new ligands acting on 5-HTRs induced extensive experimental and in silico studies oriented at the identification of determinants of compound binding to these proteins. One of the experimental techniques used with this aim is site-directed mutagenesis (SDM). It is based on the modifications of cDNA or gene promoters to introduce changes in the resulting protein (in comparison to its original sequence), such as the substitution of amino acids, insertions or deletions [17]. SDM provided great insight into the 5-HTRs mechanism of action. For example, it enabled the determination of the set of amino acids, which are important for ligand binding and receptor activation. One of the residues displaying the biggest change in ligand affinity upon amino acid replacement was aspartic acid from the third transmembrane helix—D3x32 according to the GPCRdb numbering [18,19,20,21]. These findings, together with analysis of already known ligands of 5-HTRs, led to setting up the following assumption: a compound should possess a basic nitrogen atom in its structure to have an ability to interact with the 5-HTR. In its protonated form, such nitrogen atom forms a charged-assisted hydrogen bond strongly fixing ligand in the binding site [22]. 

However, studies on the new groups of serotonin receptor ligands emerging in time revealed that the concept of high basicity was no longer applied to all compounds active towards 5-HTRs, and a number of ligands characterized by low basicity have recently been developed [23,24,25,26,27]. The non-amine structure of ligands is important in terms of developing new drug-like compounds, as it can help in getting rid of side effects. It refers especially to cardiotoxicity that is a frequent drawback of amine compounds, due to blocking of hERG potassium channels [28].

Up to 2013, the structure-based computational investigations within the 5-HTRs family were solely based on the predicted structure of the target, as no crystal structure of these protein representatives was available at that time. The first crystal structures of 5-HTRs were released in 2013 by Wacker et al. (5-HT_2B_; PDB code: 4IB4 [29]) and Wang et al. (5-HT_1B_; PDB codes: 4IAQ, 4IAR [30]). At the end of the same year, Liu et al. published another crystal construct of 5-HT_2B_ (PDB code: 4NC3) [31]. Up to now, 16 crystal structures of serotonin receptors are available, for their four subtypes: 5-HT_1B_, 5-HT_2A_, 5-HT_2B_, and 5-HT_2C_ [32]. 

In the study, a set of nonbasic ligands of the crystallized representatives of 5-HTRs was analyzed, both from ligand- and structure-based perspective with special focus on the 5-HT_2_ subfamily (5-HT_1B_R was not analyzed in detail due to a small number of known ligands with low basicity). We focused on the crystallized proteins to eliminate the uncertainty factor related to homology modeling of protein structure. The nonbasic ligands were examined in terms of their interaction schemes with the target protein and compared with their analogues with higher basicity. The study was performed for the native protein structures and for virtual mutants, in which the D3x32 residue was replaced with alanine. The investigation resulted in the determination of interactions characteristic for ligands with low basicity, which can be used in the design of new nonbasic ligands of 5-HTRs. It also extensively broadens the knowledge on nonstandard structural requirements for activity towards serotonin receptors.

## 2. Results and Discussion

### 2.1. History of 5-HT_2_R Ligands with Low Basicity

The first representatives of the nonbasic 5-HT_2_R ligand groups of the almost 800 described up to now [33] have been discovered serendipitously. The most abundant group—1,3-diaryl ureas—(see Table 1: 5-HT_2A_/Clusters 7 and 8, 5-HT_2B_/Clusters 16 and 18, 5-HT_2C_/Clusters 15 and 16) was identified by a systematic study of the impact of various indole derivatives on the serotoninergic system. The first-in-class—selective 5-HT_2C/2B_ receptor antagonist—SB200646A was uncovered in 1993 by previous consecutive structural modifications of 2-methyl-3-ethyl-5-(dimethylamino)-indole [34,35,36]. Since then, several series of analogues of the prototype SB200646A have been developed; i.e., derivatives of 1-(3-pyridylcarbamoyl)indolines [37,38,39,40,41], 1-(3-pyridylcarbamoyl)-1,2,3,5-tetrahydropyrrolo[2,3-f]indoles [42], bisaryl imidazolidin-2-ones [43], (3-methyl-5-isothiazolyl)ureas [44], diphenylureas [45] and various 1,3-biarylureas [46]. Sorafenib (Nexavar), a kinase inhibitor approved in 2007 to treat cancer, and nelotanserin (APD-125), a 5-HT_2A_R inverse agonist developed in clinical trials to treat insomnia, and Lewy body disease, are as well included in the nonbasic 5-HT_2_R ligands group bearing 1,3-diaryl urea functionality [45,47]. Nelotanserin was also a prototypic structure for series of phenethylpiperazine amides—selective 5-HT_2A_R antagonists. The 2,4-difluorophenyl urea moiety of nelotanserin was replaced with a fluorophenethylpiperazine fragment, wherein the decrease of basicity was achieved by introducing a carbonyl group into the ethyl linker [48]. 

Derivatives of diaryl sulfones (Table 1: 5-HT_2A_/Cluster 11, 5-HT_2C_/Cluster 11) are the second most abundant group of nonbasic 5-HT_2_R ligands. They are originally derived from studies on the improvement of drug-like properties of phenylsulfonyl piperidines—classical basic 5-HT_2_R ligands. The systematic structure-activity analyses of 4-fluorosulfonylpiperidines [49] or 1-sulfonylpiperidines [23] indicated that the piperidine ring’s basicity is not a prerequisite for efficient 5-HT_2A_ receptor binding. Moreover, lowering the basicity decreased the hERG (IKr) potassium channel’s unwanted affinity and improved selectivity. The replacement of central aliphatic piperidine ring by an aromatic moiety such as phenyl [26] or pyridyl [50] led to bis-aryl sulfones with even subnanomolar affinity for the 5-HT_2A_R. Another series of nonbasic diaryl sulfones (3-phenylsulfonylcycloalkano[*e* and *d*]pyrazolo[1,5-*a*]pyrimidin-2-yl)amines (Table 1: 5-HT_2A_/Cluster 11, 5-HT_2C_/Cluster 11) as 5-HT_6/2B_ receptor ligands was based on the structure of Ro-65-7674—a highly potent and selective 5-HT_6_R antagonist [50]. 

A significant part of the low-basicity 5-HT_2_R ligands has been unexpectedly discovered in multitarget ligands studies or during selectivity profiling (off-targets) of putative selective ligands. For example, in the series of agomelatine analogues, the primary targets were melatoninergic receptors MT_1_ and MT_2_ [51,52], or in the case of adenosine derivatives A_3A_R-selective agonists were transformed into moderately potent 5-HT_2B_R and/or 5-HT_2C_R antagonists (Table 1: 5-HT_2B_/Cluster 23, 5-HT_2C_/Cluster 2) [53].

Several low-basicity compounds with 5-HT_2_R affinity have been identified in natural products derived from marine organisms, for example, a group of indole alkaloids (Table 1: 5-HT_2A_/Cluster 14, 5-HT_2B_/Clusters 1 and 2, 5-HT_2C_/Clusters 14 and 17) isolated from marine sponges, i.e., aplysinopsins (from *Thorecta aplysinopsis*) [54,55,56], meridianins (from *Psammopemma* sp.) [57].

**Table 1:** Centroids of most populated clusters of nonbasic ligands of 5-HT_2A_, 5-HT_2B_, and 5-HT_2C_ receptors and their structurally related basic analogues. Compounds with overlapping cores for particular receptor subtypes share similar background color. The most basic part of each molecule is highlighted.

5-HT_2A_/Cluster 8 [37,38,39,40,41,43,45], (80 cmpds)		5-HT_2A_/Cluster 7 [45], (34 cmpds)	
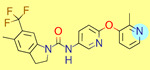 pK_a_ = 5.44	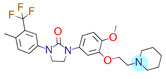 pK_a_ = 8.39 [43]	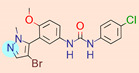 pK_a_ = 1.29	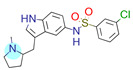 pK_a_ = 9.68 [58]
5-HT_2A_/Cluster 11 [26,50], (31 cmpds)		5-HT_2A_/Cluster 9 [48], (20 cmpds)	
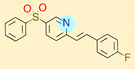 pK_a_ = 0.39	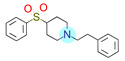 pK_a_ = 7.00 [59]	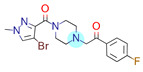 pK_a_ = 4.23	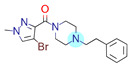 pK_a_ = 6.96 [48]
5-HT_2A_/Cluster 10 [23,26,49], (15 cmpds)		5-HT_2A_/Cluster 15 [60], (9 cmpds)	
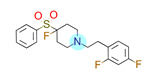 pK_a_ = 5.88	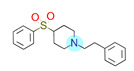 pK_a_ = 7.00 [59]	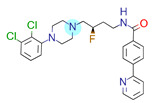 pK_a_ = 5.90	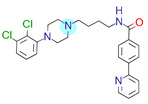 pK_a_ = 7.33 [61]
5-HT_2A_/Cluster 5 [62], (8 cmpds)		5-HT_2A_/Cluster 14 [54], (4 cmpds)	
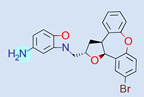 pK_a_ = 3.35	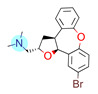 pK_a_ = 8.80 [62]	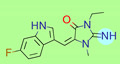 pK_a_ = 9.33	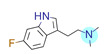 pK_a_ = 9.55 [63]
5-HT_2B_/Cluster 16 [33,34,35,36,37], (88 cmpds)		5-HT_2B_/Cluster 1, 2 [55], (33 cmpds)	
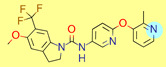 pK_a_ = 5.44	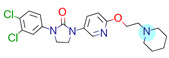 pK_a_ = 8.48 [43]	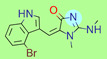 pK_a_ = 5.47	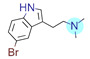 pK_a_ = 9.56 [64]
5-HT_2B_/Cluster 22 [65] (20 cmpds)		5-HT_2B_/Cluster 23 [53] (16 cmpds)	
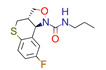 pK_a_ = −4.71		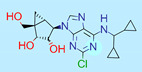 pK_a_ = 1.73	
5-HT_2B_/Cluster 18 [46] (8 cmpds)		5-HT_2B_/Cluster 10 [66] (4 cmpds)	
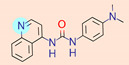 pK_a_ = 5.05		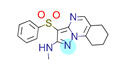 pK_a_ = 1.58	
**5-HT_2B_/Cluster 13** [67] (**4** cmpds)		**5-HT_2B_/Cluster 20** [57,62] (**2** cmpds)	
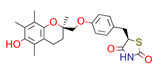 pK_a_ = −4.56		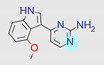 pK_a_ = 3.66	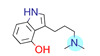 pK_a_ = 9.75 [68]
5-HT_2C_/Cluster 16 [37,38,39,40,41,42,43,45], (150 cmpds)		5-HT_2C_/Cluster 15 [44,45,46,47], (46 cmpds)	
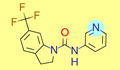 pK_a_ = 4.33	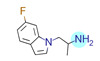 pK_a_ = 9.92 [54]	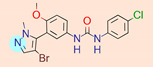 pK_a_ = 1.29	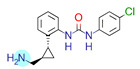 pK_a_ = 10.04 [69]
5-HT_2C_/Cluster 11 [23,49,50,59], (31 cmpds)		5-HT_2C_/Cluster 14, 17 [54,55,56], (16 cmpds)	
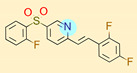 pK_a_ = 0.36	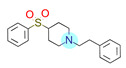 pK_a_ = 7.00 [59]	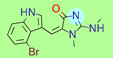 pK_a_ = 5.47	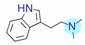 pK_a_ = 9.55 [70]
5-HT_2C_/Cluster 2 [53,71], (15 cmpds)		5-HT_2C_/Cluster 9 [51,52,72], (13 cmpds)	
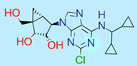 pK_a_ = 1.73		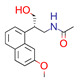 pK_a_ = −1.44	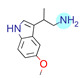 pK_a_ = 9.78 [73]
5-HT_2C_ /Cluster 3 [62], (7 cmpds)		5-HT_2C_ /Cluster 4 [68], (1 cmpds)	
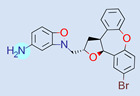 pK_a_ = 3.35	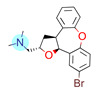 pK_a_ = 8.80 [62]	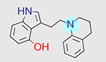 pK_a_ = 5.71	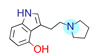 pK_a_ = 9.85 [68]

### 2.2. Datasets Analysis

At first, the comparison of the number of ligands (K_i_ below 1000 nM) with low (predicted pK_a_ below 6) and high (predicted pK_a_ above 8) basicity occurring for each target considered was carried out (Table 2).

The analysis indicates a very broad range of the fraction of basic ligands—the highest, over 20% of low-basicity compounds occur for 5-HT_2B_, whereas, for 5-HT_1B_, it is only 1% of nonbasic ligands, which refers only to eight structures. When absolute numbers are considered, the highest number of ligands with low basicity occurred for 5-HT_2C_ (329), and over 220 are available for both 5-HT_2A_ and 5-HT_2B_. The highest total number of active compounds was observed for 5-HT_2A_ (2977), and for this target, 7.66% of ligands with low basicity were detected.

**Figure 1:**
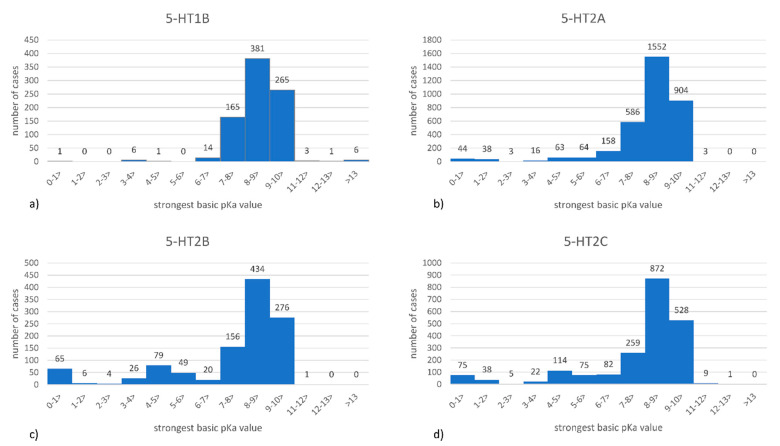
Distribution of pK_a_ values (calculated in InstantJChem as the strongest basic pK_a_) for (**a**) 5-HT_1B_, (**b**) 5-HT_2A_, (**c**) 5-HT_2B_, (**d**) 5-HT_2C_ receptor ligands.

The distribution of pK_a_ values among prepared datasets is presented in Figure 1.

The prepared histograms indicate very similar distribution of pK_a_ values among all analyzed compound sets. For each target, the highest number of compounds was characterized by the strongest basic pK_a_ in the range of (8–9>, with (9–10> range being on the second place, and (7–8> completing the top three group. For all receptors, there were only single compounds characterized by very high pK_a_ values (above 10). Interestingly, when the lower side of the pK_a_ values is considered, the lowest number of compounds fell to the pK_a_ range of (2–3>.

**Figure 2:**
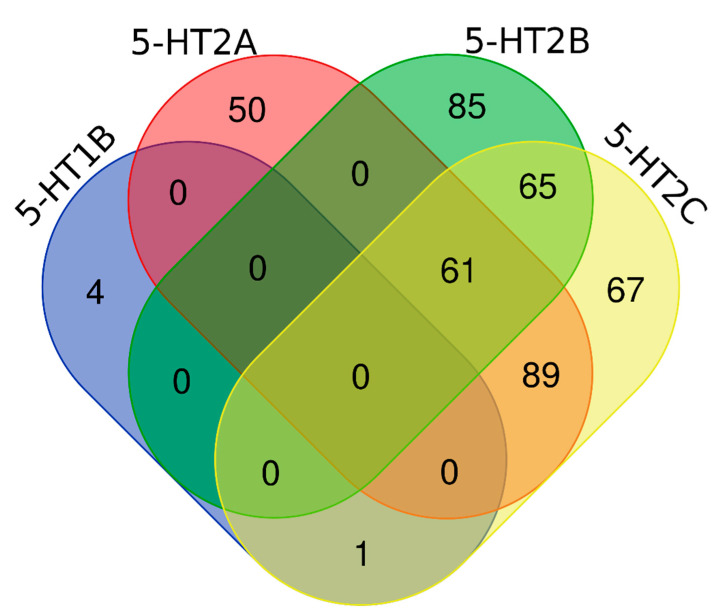
Venn diagram of non-basic ligands of 5-HT_1B_, 5-HT_2A_, 5-HT_2B_, and 5-HT_2C_ receptors.

In order to examine whether the nonbasic ligands activity is subtype specific, Venn diagrams were prepared (Figure 2).

The analysis of intersections occurring between particular ligand sets shows that although the great majority of compounds display activity only towards one receptor subtype considered, there is also a significant number of those which are active towards several 5-HTRs at the same time. There were no compounds that were active towards all four receptors considered; however, 61 compounds were simultaneously active towards 5-HT_2A_R, 5-HT_2B_R, and 5-HT_2C_R. Examples of such ligands, together with their activity profile, are gathered in Table 3. In addition, there was one compound sharing 5-HT_1B_R/5-HT_2C_R activity, 89 sharing 5-HT_2A_R/5-HT_2C_R activity, and 65 ligands which displayed dual 5-HT_2B_R/5-HT_2C_R activity.

The presented ligands display different activity and selectivity properties towards considered receptors. CHEMBL54707 and CHEMBL294030 have very low pK_a_ values (1.91 and 1.21, respectively), and are selectively active towards 5-HT_2C_R (K_i_ values of 0.5 and 1.26, respectively). Their activity towards 5-HT_2B_R is much worse, although the K_i_ values are still below 100 nM, and are equal to 10 nM for CHEMBL54707, and 50 nM for CHEMBL294030. The lowest affinity of CHEMBL54707, and CHEMBL294030 was measured for 5-HT_2A_R: 316 and 100 nM in terms of K_i_ values, respectively. The last presented compound, CHEMB240045, has no ionizable atom in its structure, and its affinity to all receptors is the lowest out of all presented compounds, with K_i_ values over 100 nM: 180 nM for 5-HT_2A_R, 170 nM for 5-HT_2B_R, and 390 nM for 5-HT_2C_R.

### 2.3. Analysis of Nonbasic Ligands by Clusters

The nonbasic ligands were also more carefully examined in terms of the structural cores, which cover the reported structures. As there is a very low number of 5-HT_1B_R ligands, the analysis was carried out only for the 5-HT_2_R subfamily. 

At first, centroids of clusters with the highest number of elements were indicated. Then, for each such element, its structurally related analogue from the set of basic ligands was determined (Table 1). When it was impossible to find an analogue in the set of compounds with pK_a_ over 8, lower pK_a_ values were allowed (in the case no basic equivalent was found, the respective cell is empty). In each case, the most basic part of a compound is circled. 

For all 5-HT_2_R subtypes, the highest populated clusters share the same core for nonbasic compounds (cluster 8 for 5-HT_2A_R, cluster 16 for 5-HT_2B_R, and cluster 16 for 5-HT_2C_R). The respective nonbasic compounds are composed of indole and pyridine moieties (terminal pyridine is the most basic part of the molecule). Their basic equivalents are built from imidazolidine, nonterminal pyridine, and terminal piperidine, responsible for high compound basicity. The difference in pK_a_ between the compared examples is ≈3.

Cluster 7 of 5-HT_2A_R nonbasic ligands, with 34 representatives, is characterized by very low predicted pK_a_ values (pK_a_ of the cluster centroid is equal to 1.29, with pyrazole as the most basic moiety). On the other hand, their basic analogues are of high basicity due to the pyrazole substitution by amino group (pK_a_ of 9.68 of the most similar compound to the cluster centroid). Over 30 representatives are also present in the cluster 11 of 5-HT_2A_R ligands. For these compounds, low basicity was obtained via the replacement of piperidine by pyridine, which significantly dropped down the compound pK_a_ values. The nonbasic properties for representatives of clusters 9, 10, 15, and 5 of 5-HT_2A_R were obtained via the introduction of electron-withdrawing (inductive withdrawing) fluorine substituents. 

Similar substitution rules are observed also for 5-HT_2B_R and 5-HT_2C_R nonbasic and basic ligand pairs; however, for 5-HT_2B_R, for as many as five analyzed clusters, the basic analogues were not found.

**Figure 3:**
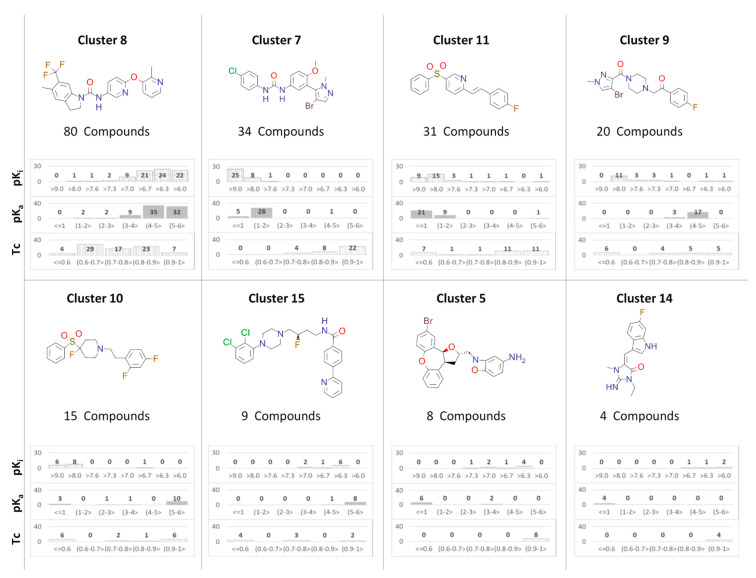
Distribution of selected compound properties (pK_i_, predicted pK_a_ and Tc values towards cluster centroid) for the most populated clusters of non-basic 5-HT_2A_R ligands.

In order to examine in more detail the properties of compounds belonging to each cluster, histograms of their property distributions were prepared (Figure 3). We focused on the compound activity (expressed as pK_i_), basicity (expressed as predicted pK_a_ values), and structural consistency within cluster (expressed as the Tanimoto coefficient (Tc) values [74] towards cluster centroid). The prepared histograms indicate that there is high variation in the distribution of examined properties of 5-HT_2A_R ligands. For example, for cluster 8, the compound pK_i_ range is from 6.0 to 7.0 for the majority of ligands (corresponding to 100–1000 nM K_i_), which can be described as moderate activity, whereas most representatives of clusters 7 and 11 have pK_i_ above 9 (which means K_i_ below 1 nM). Moreover, compounds from clusters 7 and 11 are characterized by significantly lower basicity (pK_a_ of the majority of them was below 2), than representatives of cluster 8 (pK_a_ mostly from 4–6). Out of these three highest populated clusters, cluster 7 is most structurally consistent (most of the compounds have Tc values towards cluster centroid above 0.9), although for cluster 11, most of the compounds are also structurally related to a high extent to the respective centroid (Tc above 0.8). However, in cluster 11, there are seven compounds which are more diversified from the rest of the cluster representatives, with Tc towards cluster centroid below 0.6. Cluster 8 has the highest number of members, but they are also the most diversified (over half of the compounds have Tc towards centroid below 0.7). 

For the rest of the clusters, the situation varies, although usually most of the cluster members fall within similar activity and basicity range. Cluster 9 is composed of high-activity ligands (pK_i_ > 8), with pK_a_ between 4 and 5, cluster 10 is formed by 15 compounds of even higher 5-HT_2A_R activity (six compounds with pK_i_ above 9), but also with slightly higher basicity (pK_a_ between 5–6). Cluster 15 is characterized by lower activity (pK_i_ below 7 for all cluster representatives) and similar basicity to cluster 10. Clusters 9, 10, and 15 are also characterized by relatively high structural diversity. On the other hand, clusters 5 and 14 are very consistent in terms of compound structure, but their representatives vary in terms of the 5-HT_2A_R affinity. Moreover, clusters 5 and 14 gather compounds with very low basicity (the great majority of them has predicted pK_a_ below 1).

### 2.4. Docking Analysis—By Clusters

**Figure 4:**
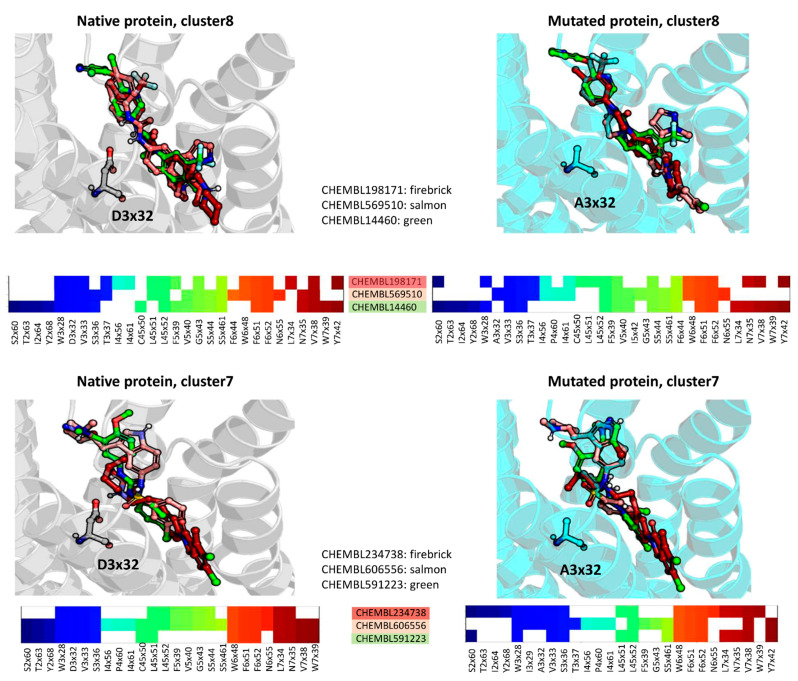
Docking results of centroids of non-basic compound clusters of 5-HT_2A_R ligands with the higher number of representatives (cluster 8 and cluster 7, depicted in green), together with the structurally related basic compounds (depicted in shades of red). In each case, the residue at 3x32 position is depicted in sticks (D and A for native and mutated receptors, respectively). Below each ligand-protein complex, occurring contacts are presented in form of the interaction matrix.

The clusters of nonbasic ligands were analyzed in terms of their interaction schemes with the target proteins, both native and virtually mutated (Figure 4). The figure presents the top poses obtained for native and mutated 5-HT_2A_R. For cluster 8 and cluster 11, the occupancy of 5-HT_2A_R binding site by ligands in native and mutated receptor is almost the same, whereas for cluster 7, the compounds in the mutated receptor tend to shift towards the 5th transmembrane helix (5TM). For both clusters, their centroids do not interact with A3x32 (although the contact with D3x32 is present). Upon D3x32A mutation, also CHEMBL198171, being a basic analogue of cluster 8 centroid, lost interaction with residue in this position. On the other hand, CHEMBL569510 (another basic analogue for cluster 8 representatives) does not make contact with helix 7 in the D3x32A mutated receptor.

In cluster 7, the differences were a little bit different. Basic compounds, CHEMBL234738 and CHEMBL606556, behaved differently when shifting from native to mutated receptor. The former ligand changed its conformation in the mutated receptor in such a way that it lost contact with the TM2, and some residues from TM7. On the other hand, CHEMBL606556 lost interaction with TM5, but gained contact with TM2. The most indicated difference between basic and nonbasic compounds from cluster 7 is the lack of interaction with TM5 by the nonbasic ligand in the native receptor and no contact with A3x32 in the mutated 5-HT_2A_R. 

It is worth noting that mutation of the D3x32 residue seems to have higher impact on the docking pose of basic compounds than ligands with low basicity. The docking results also indicate that the lack of strong hydrogen bond with D3x32 does not always entail the significant change in the compound conformation and the network of other ligand–protein contacts is strong enough to keep the ligand in its position adopted for the native protein.

### 2.5. Docking Studies—Whole Dataset Analysis

**Figure 5:**
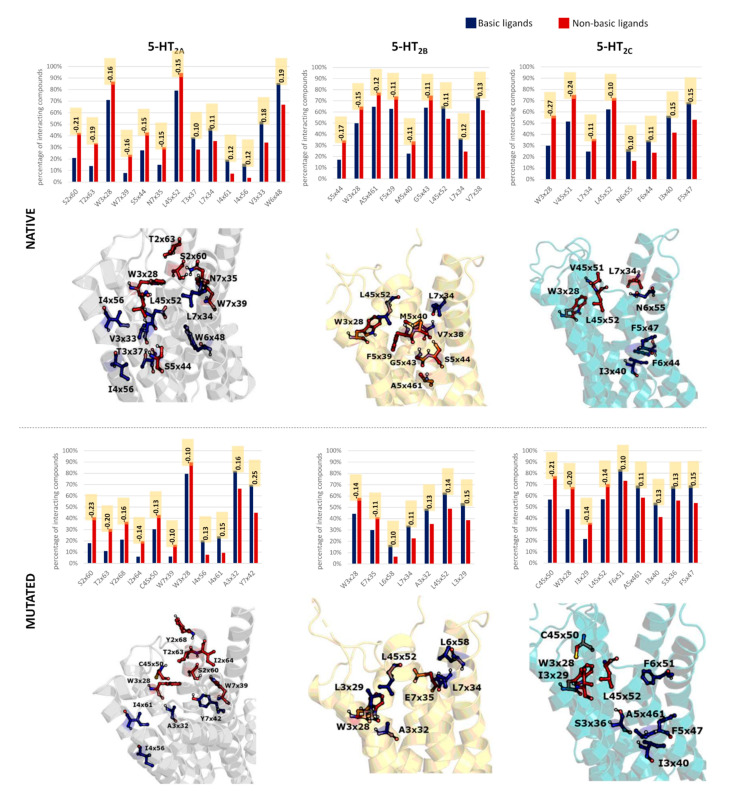
Comparison of interaction frequencies with basic and non-basic compounds of selected amino acids (positions with the highest differences in the interaction frequencies are presented), together with their indication in the respective crystal structures—5-HT_2A_: 6A94, 5-HT_2B_: 4IB4, 5-HT_2C_: 6BQH. Differences in the interaction frequencies are depicted in orange boxes.

Detailed analysis between the frequency of interactions of ligands with particular residues was carried out (Figure 5). In all cases, residues with the highest difference (above 0.1) in the interaction frequency between the compared compound groups are indicated.

For the native proteins, the highest number of discriminating positions between basic and nonbasic compounds occurred for 5-HT_2A_R (13 positions). Some of the positions are also the most differentiating when D3x32A mutated 5-HT_2A_R is considered (such as S2x60, T2x63, W3x28, W7x39, etc.), but some are unique for the nonmutated receptor form (such as W6x48 which interacted with 20% less nonbasic compounds in comparison to the set of basic ligands). Another residue with high preference to interact with basic compounds is V3x33 (18% difference in the interaction frequency between basic and nonbasic compounds). On the other hand, the highest preference for contact with nonbasic ligands of 5-HT_2A_R was for residues from TM2 (S2x60 and T2x63, ≈20% more frequent than basic compounds) for both native and mutated receptors.

In the mutated 5-HT_2A_R, the highest preference for basic ligands was observed for Y7x42 (25%). Residues from TM7 were also preferably interacting with basic ligands of 5-HT_2B_R (L7x34 and V7x38 for native receptor and L7x34 for the mutated 5-HT_2B_R). Both 5-HT_2A_R and 5-HT_2B_R were also interacting preferably with A3x32, when mutated receptor forms were taken into account.

The nonbasic ligands of 5-HT_2C_R interacted much more frequently with residues from ECL2 and W3x28 for both native and mutated receptor form (W3x28 in the native 5-HT_2C_R was the residue with the highest discriminative power: 27% difference in the interaction frequency between basic and nonbasic compounds).

## 3. Materials and Methods

The compound datasets were prepared on the basis of the ChEMBL database v26 [33]. All K_i_-based records related to 5-HT_1B_, 5-HT_2A_, 5-HT_2B_, and 5-HT_2C_ receptors were obtained. As we wanted to focus only on active compounds, all records with K_i_ values above 1000 nM were filtered out. The strongest basic pK_a_ value was determined for each compound using InstantJChem [75]. The ligands were divided into basic and nonbasic sets, by applying the pK_a_ threshold equal to 8 and 6, respectively (pK_a_ < 6: nonbasic compounds; pK_a_ > 8: basic compounds). The respective ligand sets were clustered in Canvas using MOLPRINT2D [76] for compounds representation, Tc values were used for measurement of the distance between compounds, and the Kelley criterion [77] was used for the determination of the number of clusters.

The compounds were docked to the respective crystal structures of 5-HTRs. The compounds were prepared for docking using the tool from the Schrödinger Suite–LigPrep [78]: protonation states generated at pH 7.4 +/− 0.0; a maximum of four stereoisomers per compound was generated, and other settings remained at the default. The crystal structures for docking were fetched from the PDB database, and the following structures were used in the study—5-HT_1B_: 4IAR [30], 5-HT_2A_: 6A94 [79], 5-HT_2B_: 4IB4 [29], 5-HT_2C_: 6BQH [80]. The coordinates were prepared for docking using tools from the Schrödinger package (Protein Preparation Wizard). The D3x32 residue (A3x32 in mutated receptors) constituted the grid center in each case, and the grid size was set to 23 Å. The docking was performed in the extra precision mode in Glide [81] and for the resulting ligand–protein complexes, the structural interaction fingerprints (SIFts) [82,83] were generated using Schrödinger tools. 

## 4. Conclusions

In the time of highly desirable studies on the search for structurally new ligands, we carefully examined the low basicity as a feature of atypical serotonin receptor ligands. Getting rid of the requirement of the presence of basic nitrogen in the compound structure in order to provide its activity within the 5-HT system does not only allow exploration of new fragments of chemical space, but can also be helpful when attempting to eliminate side effects. In the study, the ligands of crystallized representatives of serotonin receptors underwent examination. In particular, we focused on the compounds active towards 5-HT_2_ receptors, due to their relatively high number. At first, detailed analysis of structures of ligands with low basicity was carried out, and several clusters of compounds were identified within each receptor subtype, together with their basic analogues. Clusters with the highest number of representatives were studied via docking, and compounds with low basicity were compared with the typical basic ligands in terms of their interaction with the target protein. Then, similar comparisons were made for the whole datasets of basic and nonbasic ligands, for both native and D3x32A mutated form of receptors. Such study allowed for detection of positions which are discriminative for these two ligand groups and which should be carefully considered when designing new atypical ligands of 5-HTRs. The outcome of this study can be of great help during the 5-HTR ligands development, especially with atypical, nonbasic structure.

## Figures and Tables

**Table 2:** Comparison of the number of records with low (pK_a_ < 6) and high (pK_a_ > 8) basicity for the considered targets. In brackets, the fraction of basic ligands among all active compounds is provided.

	5-HT_1B_	5-HT_2A_	5-HT_2B_	5-HT_2C_
Low basicity	8 (1.04%)	228 (7.66%)	229 (22.50%)	329 (17.06%)
High basicity	584 (75.75%)	2005 (67.35%)	613 (60.22%)	1258 (65.25%)
Total number of ligands	771	2977	1018	1928

**Table 3:** Examples of nonbasic ligands displaying simultaneous activity towards 5-HT_2A_, 5-HT_2B_, and 5-HT_2C_ receptors, together with their affinities expressed as K_i_.

Ligand Structure	CHEMBLID	pK_a_	5-HT_2A_R K_i_ [nM]	5-HT_2B_R K_i_ [nM]	5-HT_2C_R K_i_ [nM]
* 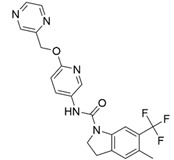 *	CHEMBL54707	1.91	316	10	0.5
* 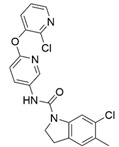 *	CHEMBL294030	1.21	100	50	1.26
* 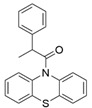 *	CHEMBL240045	No ionizable atom	180	170	390

## Data Availability

Not applicable.

## Abbreviations

5-HTRs	serotonin receptors
CNS	central nervous system
GPCRs	G protein-coupled receptors
TMDs	transmembrane domains
ICL	intracellular loop
ECL	extracellular loop
SDM	site-directed mutagenesis
Tc	Tanimoto coefficient
SIFt	Structural Interaction Fingerprint
